# Shear influence on colloidal cluster growth: a SANS and USANS study

**DOI:** 10.1107/S1600576723006726

**Published:** 2023-08-25

**Authors:** Chris Muzny, Liliana de Campo, Anna Sokolova, Christopher J. Garvey, Christine Rehm, Howard Hanley

**Affiliations:** a Applied Chemicals and Materials Division, National Institute of Standards and Technology, Boulder, CO 80305, USA; b Australian Nuclear Science and Technology Organisation, Locked Bag 2001, Kirrawee DC, NSW 2232, Australia; c Forschungs-Neutronenquelle, Heinz Maier-Leibnitz (FRM II), Technische Universität München, Lichtenbergstraße 1, Garching, 85748, Germany; d Applied Mathematics, Research School of Physics, Australian National University (ANU), Canberra, ACT 2600, Australia; Brazilian Synchrotron Light Laboratory, Brazil

**Keywords:** colloidal silica, retarded gelation, applied shear, viscosity, time-dependent phenomena, small-angle neutron scattering, SANS, ultra-small-angle neutron scattering, USANS, micrometre scale, structure factor derivation, volume fraction

## Abstract

This study examines the time evolution of silica/water clusters where the formation of a gel network from unitary silica particles is interrupted by a simple Couette shear field. The aim is to enable the general understanding of this simple system by examining the microscopic basis for the changes in viscosity by providing structural inputs from small-angle scattering for a simple theoretical model. The experimental system is an 8.3 nm particle silica solution (Ludox) where the gelation has been initiated by lowering the pH in a Couette cell providing a constant shear rate of 250 s^−1^. A unified small-angle neutron scattering (SANS) and ultra-small-angle neutron scattering (USANS) procedure is described to measure the scattered intensity in a wavevector range of 3 × 10^−4^ ≤ *q* (nm^−1^) ≤ 3.1 × 10^−1^, probing structural changes over a broad range of length scales from the nanometre to the micrometre. Scattering data provide a new means of better understanding the behaviour of colloidal clusters when subjected to an external applied shear over a continuous time sequence after gel initiation; a fit of the time-dependent scattered intensity leads to an estimation of the cluster’s effective volume fraction and size as a function of time. A reductionist theoretical basis is described to predict the time-dependent viscosity behaviour of the sheared colloidal suspension gel-initiated cluster growth from the volume fraction of the clusters.

## Abstract 2

This study examines the time evolution of microscopic structure in a model industrial process, a sol of gel-initiated silica particles in a simple Couette shear field, using small-angle neutron scattering SANS) and ultra-small-angle neutron scattering (USANS) over a large range of scattering vector magnitudes/length scales. Comparison of the experimentally derived parameters with a simple theoretical model provides additional insight.

## 1. Introduction

The coordination between industrial and academic studies of colloidal gels is an example of how the commercial performance of a product, or opportunities to develop a new product, rely on having background chemistry and physics data for each step throughout the manufacturing process (Bergna, 2005 ▸; Ramsay *et al.*, 2005 ▸; Sobotka, 1984 ▸). In particular, knowledge of the properties and behaviour of silica gels is essential since such gels are the ingredients of a wide variety of commercial products, of which cosmetics, pharmaceuticals, paints and food items are only a few. The significance of gels to the food industry is a typical example that has been discussed at length (Tabilo-Munizaga & Barbosa-Cánovas, 2005 ▸; Saha & Bhattacharya, 2010 ▸; van Heijkamp *et al.*, 2010 ▸).

Much of the understanding of how colloidal gel networks form is extracted from techniques that probe a system using non-destructive radiation (light, X-rays and neutrons; Lindner & Zemb, 2002 ▸) and oscillatory rheology (Bantawa *et al.*, 2023 ▸). Rheological investigations are indirect, while scattering techniques provide direct structural evidence of the network during its formation (Teixeira, 1986 ▸). In our case, the objective was to investigate the nanometre- and micrometre-range organization of a gel-initiated colloid subjected to a constant external force, in this work an external applied shear rate, continuously over time. The coupling between the two approaches can be most informative (Eberle & Porcar, 2012 ▸; Weigandt *et al.*, 2009 ▸; Gadala-Maria & Acrivos, 1980 ▸; Rueb & Zukoski, 1997 ▸; Hanley *et al.*, 1999 ▸; Drabarek *et al.*, 2000 ▸; King *et al.*, 2007 ▸; Kalman & Wagner, 2009 ▸) but, for this task, a neutron radiation source is particularly appropriate because a radiation probe allows the sample’s structure to be tracked directly over time. We refer to Eberle & Porcar (2012 ▸) for a definitive survey of the various options: sample multiple scattering can restrict the utility of light; rheo–X-ray procedures are very much an emerging field (Panine *et al.*, 2002 ▸; Hyun *et al.*, 2008 ▸; Philippe *et al.*, 2011 ▸; Ben Messaoud *et al.*, 2020 ▸; Narayanan *et al.*, 2020 ▸; Gibaud *et al.*, 2020 ▸); small-angle neutron scattering (SANS) and ultra-small-angle neutron scattering (USANS) procedures have a practical advantage largely because the techniques are well established and relatively straightforward; and lastly, the neutron apparatus allows online equipment, in this case a rheometer, to be robust.

Time-dependent shear-influenced evolution studies of colloidal behaviour are relatively scarce (Gadala-Maria & Acrivos, 1980 ▸; Hanley *et al.*, 1999 ▸; Muzny *et al.*, 1994 ▸; Laun *et al.*, 1992 ▸). The first attempt to probe a sheared system over the nanometre and micrometre ranges was reported by Muzny *et al.* (1999 ▸), who merged gelled silica SANS results with USANS data using the technique developed by Agamalian and co-workers (Schaefer & Agamalian, 2004 ▸; Bhatia, 2005 ▸). The result from the USANS experiment was satisfactory but gave no time evolution information because the silica sample was pre-sheared at 500 s^−1^. More recently, however, de Campo *et al.* (2019 ▸) measured the time-dependent shear-influenced behaviour of a gel-initiated silica solution over the micrometre scale. The approach taken in the present paper was to cover both the nanometre and micrometre regimes and thus continue our investigation of the time-dependent behaviour of a gel-initiated silica solution subjected to an applied shear rate. Of the several areas for study one was of current relevance to us, namely, how best to model shear-influenced silica cluster development over the time span between the sample’s gel initiation and a steady state. The paper addresses this and proposes an experimental procedure. The necessary scattered intensity data were extracted using the SANS and USANS facilities in operation at the Australian Centre for Neutron Scattering of the Australian Nuclear Science and Technology Organisation (ANSTO, Lucas Heights, Australia). Silica solutions (Iler, 1979 ▸) were gel-initiated and subjected to an applied shear rate of 250 s^−1^.

## 2. Methods and materials

### 2.1. Colloidal silica gelation

The sample was a stock aqueous suspension of commercial-grade Ludox AS-30 colloidal silica (SiO_2_) at pH ≃ 9.6 supplied by W. R. Grace & Co., Columbia, USA.^1^ The suspension had 31 mass% SiO_2_, corresponding to a silica volume fraction ϕ = 0.17, with an SiO_2_ density of 2.2 g cm^−3^. The spheres have a nominal diameter of σ = 8.3 nm with a polydispersity estimated at 20%. Gelation was initiated at time zero by lowering the pH of the sol to ∼8 with added HCl, as reported in our previous work which includes sheared and unsheared systems (Hanley *et al.*, 1999 ▸).

### 2.2. Equipment

#### 2.2.1. Rheometer

The sample cell used was a commercial MCR-500 rheometer (Anton Paar GmbH, Graz, Austria) constructed with a home-built Couette shear cell consisting of two concentric quartz cylinders, with an external cylinder diameter of 10 cm and a 0.5 mm gap between the cylinders. In Cartesian coordinates, the constant applied shear rate was with the neutron beam incident along the *y* axis and the flow velocity of the sample *u_x_* in the *x* direction. The beam diameter was 1 cm. The rheometer setup followed a standard procedure (Hanley *et al.*, 1999 ▸; de Campo *et al.*, 2019 ▸) and the viscosity of water was measured periodically to verify that the cell was correctly calibrated.

#### 2.2.2. SANS (Bilby) and USANS (Kookaburra)

Pinhole SANS data were obtained from ANSTO’s instrument Bilby operating in monochromatic mode (Sokolova *et al.*, 2019 ▸). We measured the time-dependent scattered intensity data *I*(*q*, *t*) with Bilby in monochromatic mode at a wavelength of λ = 0.5 nm. The detector bank was set to cover a simultaneous wavevector range of 4.7 × 10^−2^ ≤ *q* (nm^−1^) ≤ 1.4, given *q* = (4π/λ)sin(2θ/2), with 2θ the scattering angle.

The USANS Kookaburra instrument has been described previously (Rehm *et al.*, 2013 ▸, 2018 ▸). Kookaburra was set with a neutron wavelength λ = 0.474 nm to measure the slit-smeared scattered intensity in a point-by-point fashion, by rotating an analyser crystal and monitoring the scattered beam intensity, as is typical of Bonse–Hart-type instruments (Bonse & Hart, 1965 ▸). Previously it was verified that significant multiple scattering effects were absent from these samples (de Campo *et al.*, 2019 ▸). Details of the general experimental procedure have been given by de Campo *et al.* (2019 ▸).

### 2.3. Data acquisition

#### 2.3.1. SANS on Bilby

The rheometer was set to measure the viscosity, η, of the sample subjected to an applied shear rate 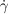 of 250 s^−1^. It was programmed to record the value of the viscosity at one-minute intervals throughout the duration of a given run using the *RheoPlus* software (Anton Paar). Immediately after gelation initiation with HCl to adjust the pH to ∼8, the sample was mixed using a magnetic stirring bar for approximately 5 s. This sample solution was loaded into the Couette cell. The cell was sealed with a water-filled solvent trap to prevent evaporation and set in the neutron beam path such that the incident beam illuminated the centre of the Couette cell (*y* direction). After a roughly two-minute delay determined by the sample preparation and facility safety procedures, the rheometer and Bilby data acquisition were activated simultaneously. The scattered intensity time-dependent measurements *I*(*q*, *t*) were averaged over 5 min intervals. The *I*(*q*, *t*) data were obtained from the raw isotropic 2D detector images using data reduction following the procedure of Sokolova *et al.* (2019 ▸) using the *Mantid* software (Arnold *et al.*, 2014 ▸).

#### 2.3.2. USANS on Kookaburra

The sample preparation was the same as for Bilby, but on Kookaburra, due to the Bonse–Hart system and the point-by-point measurement, it is not possible to measure intensities simultaneously over a wavevector range. Accordingly, Kookaburra’s settings were selected to record data at four points (*q* values) chosen to cover an order of magnitude in intensity: these points are listed in Table 1 ▸. Further, because the scattered intensity change was most rapid at the start of the experiment, it was decided to extract the USANS time-dependent data measured from four independent runs, each associated with an initial *q* value and a separate silica gelation process. Each run was divided into two segments, designated segment *A* (single point counted for 1000 counts or 2 min) with one point from Table 1 ▸, and segment *B* (cycle of 8.5 to 12.5 min over four points counted for 1000 counts or 5 min) with *q* values continuously cycled from Table 1 ▸.

The experiment proceeded by first measuring the *q*_1_ intensity as a function of increasing time over about four hours after the sample’s gel initiation (segment *A*, run 1). The measurements were then extended to cover about 27 h with the sample cycled over the four *q* values (segment *B*, run 1). The procedure was repeated to measure the equivalent intensity data for wavevectors *q*_2_ to *q*_4_ treated separately. Segment *B*, run 2 measured the intensities over 25 h to confirm that the data were consistent with those of run 1. It was therefore considered sufficient to limit the results for segment *B*, runs 3 and 4 to about one hour. All the intensity data were reduced to the absolute scattering cross section by applying standard procedures (Kline, 2006 ▸) using the *Gumtree* software (Xiong *et al.*, 2017 ▸).

## 3. Results and discussion

### 3.1. Viscosity

**Figure 1:**
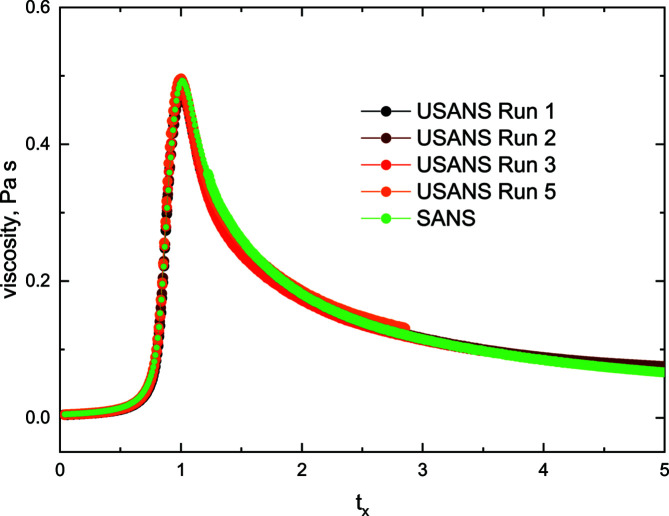
The viscosity versus time behaviour during one SANS and four USANS measurements, with the gelling silica samples subjected to a shear of 250 s^−1^. The time *t_x_* is the experimental time scaled with respect to *t*_max_ of the maximum viscosity peak (*t_x_* = time/*t*_max_).

Under normal conditions, a gel-initiated colloidal silica solution would progress until a time is reached – loosely designated the gel time – when the solution morphs into a network-generated gel (Drabarek *et al.*, 2000 ▸; Wang *et al.*, 2014 ▸; Mohraz & Solomon, 2005 ▸; Shih *et al.*, 1990 ▸) with an effective volume fraction defined as unity, the volume occupied by aggregates, and, in principle, an infinite viscosity. When subjected to an applied shear rate, however, the solution is prevented from gelling and the viscosity increases to reach a peak at a time *t*_max_ close to the equivalent gel time. The viscosity then decreases to approach an asymptotic value (Fig. 1 ▸). We observed macroscopically by inspection, but also from viscosity measurements when the shear was interrupted and restarted, that the system will cease to flow and will form a gel at any time when the applied shear is removed.

It is convenient to record the time-dependent behaviour of a sheared gelling system in terms of a reduced time *t_x_* scaled with respect to *t*_max_ and defined as *t_x_* = 1.0 at the shear-induced viscosity peak (*t*_max_). The behaviour of many shearing runs under different conditions can be consolidated and checked for reproducibility using this constraint (Hanley *et al.*, 2007 ▸, 1999 ▸; de Campo *et al.*, 2019 ▸). Fig. 1 ▸ displays the viscosity behaviour of the gelling silica as a function of *t_x_*. The actual times for different runs corresponding to *t*_max_ were 110 ± 5 min.

### 3.2. SANS

The time-dependent SANS intensities, although averaged over 5 min intervals, are recorded as continuous in *q*. In order to blend them with the USANS results and hence define a wide *q* range, it was convenient to identify intensities at selected *q* values (Table 2 ▸).

**Figure 2:**
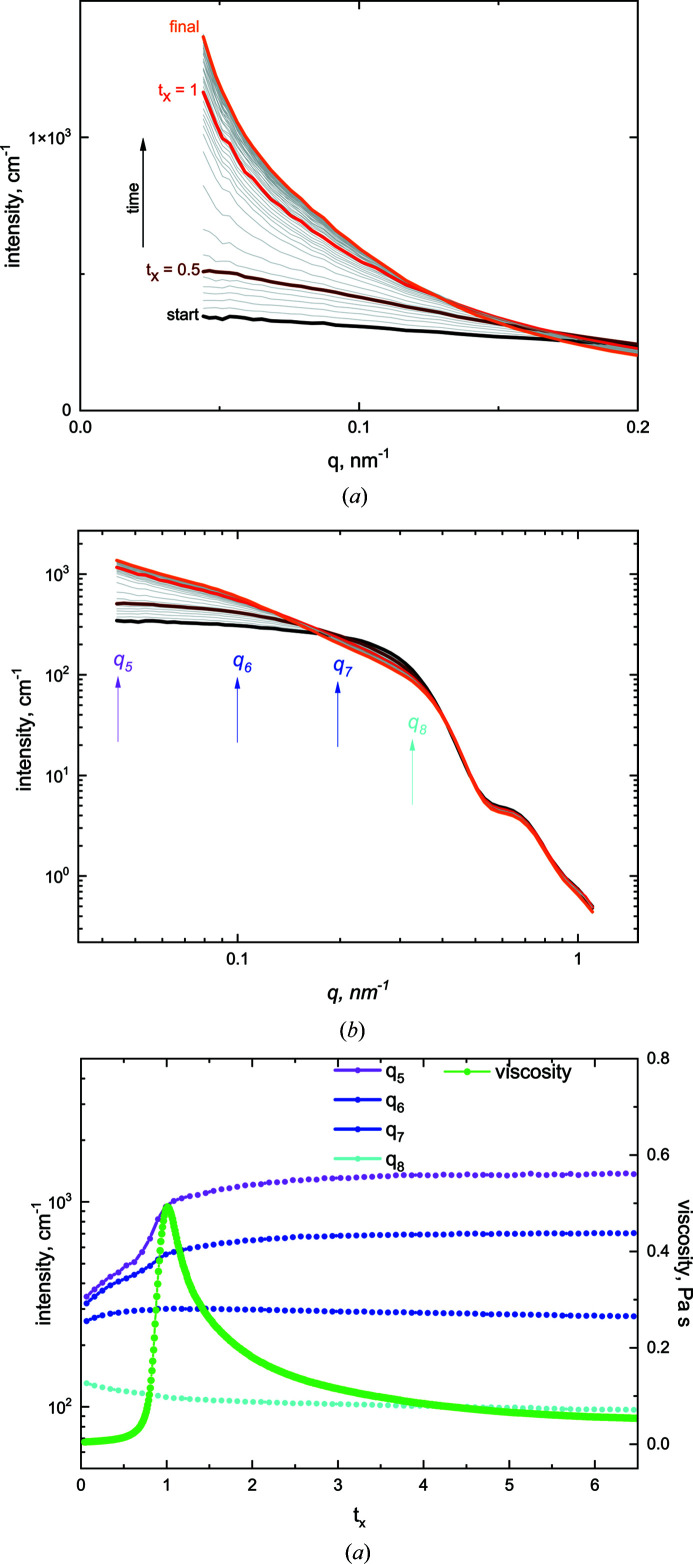
(*a*) and (*b*) SANS data with the time evolution of the scattered intensity for the system over the SANS *q* range on (*a*) a linear and (*b*) a logarithmic scale. The curves indicate that the intensity increases after gel initiation until the system is essentially stable. (*c*) Plots of the corresponding intensities shown by arrows in panel (*b*) together with the viscosity.

The results shown in Figs. 2 ▸(*a*) and 2 ▸(*b*) are the linear and logarithmic scale plots, respectively, of *I*(*q*, *t*) at times between a start time (of the order of minutes after the initiation of gelation) and a final time when the shearing system was effectively stable, at *t*_*x*_ ≃ 2.0 [Fig. 2 ▸(*c*)]. Fig. 2 ▸(*c*) shows the variation in the scattered intensity as a function of *t_x_* at four representative wavevectors, *q*_5_ to *q*_8_ of Table 2 ▸, together with the viscosity. The *q*_5_ curve in particular indicates the correlation between the viscosity peak (*t*_*x*_ = 1.0) and an increase in the intensity (Drabarek *et al.*, 2000 ▸; de Campo *et al.*, 2019 ▸).

### 3.3. USANS

**Figure 3:**
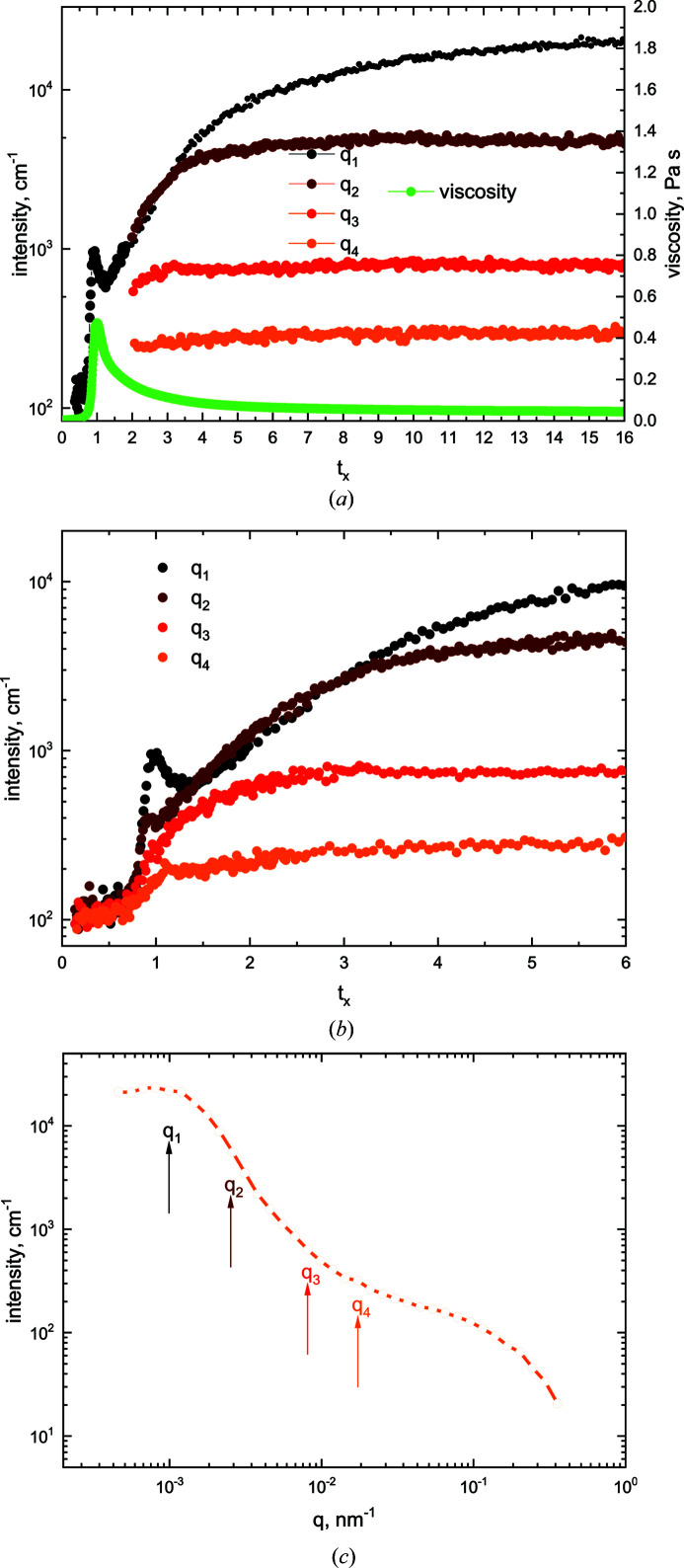
Complete (*a*) and zoomed short-timed (*b*) USANS data for selected *q* values on the rocking curve as a function of normalized time. (*c*) The entire rocking curve at steady state at *t_x_* ≃ 16. The time-dependent shear viscosity is included in panel (*a*) for reference.

Table 1 ▸ gives the numeric values for the *q* values used in Fig. 3 ▸. Fig. 3 ▸(*a*) shows the *I*(*q*, *t*) versus *t*_*x*_ curve at *q* = 1.0 × 10^−3^ nm^−1^ for a run time of ∼4.12 h (segment *A*, run 1) and the subsequent curves generated by cyclic *q* sampling over ∼27 h (segment *B*, run 1). The associated viscosity curve is also plotted at the bottom of Fig. 3 ▸(*a*). The *I*(*q*, *t*) cross plots for the four *q* values (runs 1–4) are shown in Fig. 3 ▸(*b*). The data recorded in Fig. 3 ▸(*a*) indicate that the gelling system was close to stability after a run time of *t*_*x*_ ≃ 16. Thus, a scan over the full USANS *q* range was attempted: a run over 2 h 45 min yielded acceptable results, plotted in Fig. 3 ▸(*c*).

### 3.4. Further observations on gelation

We have noted (Section 3.1) that the system will gel (visually a non-flowing system) at any time when the applied shear is removed. Figs. 2 ▸ and 3 ▸ show that the scattered intensity measurements over the nanometre and micrometre scales will, like the viscosity, approach a stable shear-influenced steady state. How long, in laboratory time, this state will remain stable is not confirmed, but it is at least of the order of days. If, however, the applied shear is disconnected, the system will gel. Both tactile and visual observations verify this result. We thus argue that the action of the applied shear is to retard network formation: the network will reform on removal of the applied shear.

How the time-dependent viscoelastic properties of this externally sheared silica gel differ from those of its unsheared equivalent has been discussed by Drabarek *et al.* (2000 ▸). In that work a silica solution was gelled by lowering the pH and the gel’s viscoelastic properties were measured. The procedure was repeated with a gel-initiated sample subjected to a shear rate of 500 s^−1^ for 4 h. The shear was turned off and the equivalent viscoelastic properties also measured. The different moduli of the gels clearly indicated that the two gels differed, even though they were from the same source. We refer to that work for a detailed interpretation of these results.

### 3.5. Steady-state SANS and USANS data

**Figure 4:**
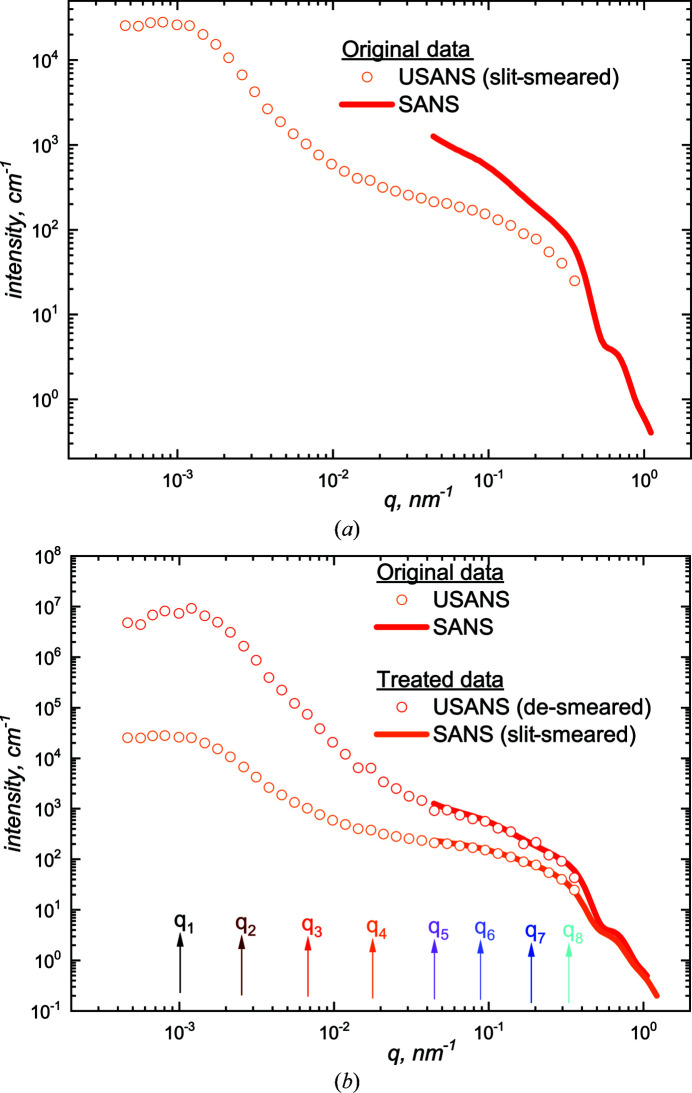
(*a*) SANS and USANS data of the shear-stabilized sample sheared at 250 s^−1^. (*b*) The curves with both the SANS and USANS data merged. The data of the lower curve are of special interest: see text. The eight *q* values listed in Tables 1 and 2 are marked.

The respective SANS and USANS curves for the samples that have reached a steady-state viscosity and exhibit no changes in scattering patterns, stabilized under the shear in terms of bulk property, viscosity and microscopic structure, are shown in Fig. 4 ▸(*a*). There is significant wavevector overlap but the different instrument smearing conditions do not allow the sets to fit visually. It is necessary either to combine the SANS data with de-smeared USANS data or to smear the SANS data with the same effective slit-height correction as the USANS data; both alternatives are acceptable. The time-dependent USANS data are of special interest here but cannot properly be de-smeared given only the four *q* values of Table 1 ▸. For consistency, therefore, the SANS data were slit-smeared with the Kookaburra slit height of 0.586 nm^−1^ (Rehm *et al.*, 2018 ▸) to yield the combined SANS and USANS intensities displayed in Fig. 4 ▸(*b*).

### 3.6. Scattering data modelling and analysis

#### 3.6.1. Static intensity USANS and SANS

The normalized stable-state intensity data [Fig. 4 ▸(*b*)] covering the full wavevector range [3 × 10^−4^ ≤ *q* (nm^−1^) ≤ 3.1 × 10^−1^] and allocated the reduced time of *t_x_* = 16 [Figs. 1 ▸ and 3 ▸(*a*)] are first considered. We have used this expression previously (Butler *et al.*, 1996*b* ▸; de Campo *et al.*, 2019 ▸; Hanley *et al.*, 2007 ▸) and the full justification for its use is described elsewhere (de Campo *et al.*, 2019 ▸). The fitting equation is written as

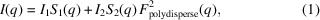

where *S*_1_(*q*) is the structure factor representing the total scattering in the low-*q* range, *S*_2_(*q*) is the structure factor accounting for the short-range correlations of the silica spheres of the stock solution, 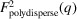 is the form factor that describes the shape of the silica particles over the wavevector range, and *I*_1_ and *I*_2_ are weighting parameters for the low-*q* and high-*q* regimes, respectively. They effectively describe the aggregation of elementary Ludox particles into larger aggregates. The equation can be simplified because the contribution of *S*_2_(*q*) was found to be effectively constant if *q* < 0.31 nm^−1^. Hence, *I*_2_ *S*_2_(*q*) is replaced by *I*_2_, where *I*_2_ is considered the weighting parameter for 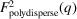 . The equation thus becomes

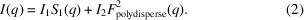

The *S*_1_(*q*) structure factor was chosen to be the empirical expression proposed by Butler *et al.* (1996*a* ▸,*b* ▸) [see also Muzny *et al.* (1994 ▸, 1999 ▸)], which follows that of Furukawa (1985 ▸) but adapted to fit the shear-influenced intensity curve previously reported,

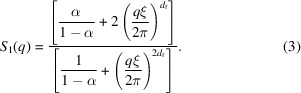

The expression is based on the experimental observation that the scattered intensity of a gel-initiated silica sample peaks (Muzny *et al.*, 1999 ▸). The peak results from the spatial correlations between low-value wavevector particle clusters growing after gel initiation. α is the value of *S*_1_(*q*) at *q* = 0, ξ is a characteristic length and *d*_f_ is a power law exponent. It is noted that, after the initial fit of the steady-state *I*(*q*) curve, α is treated as a constant for the *I*(*q*, *t*) plots.

**Figure 5:**
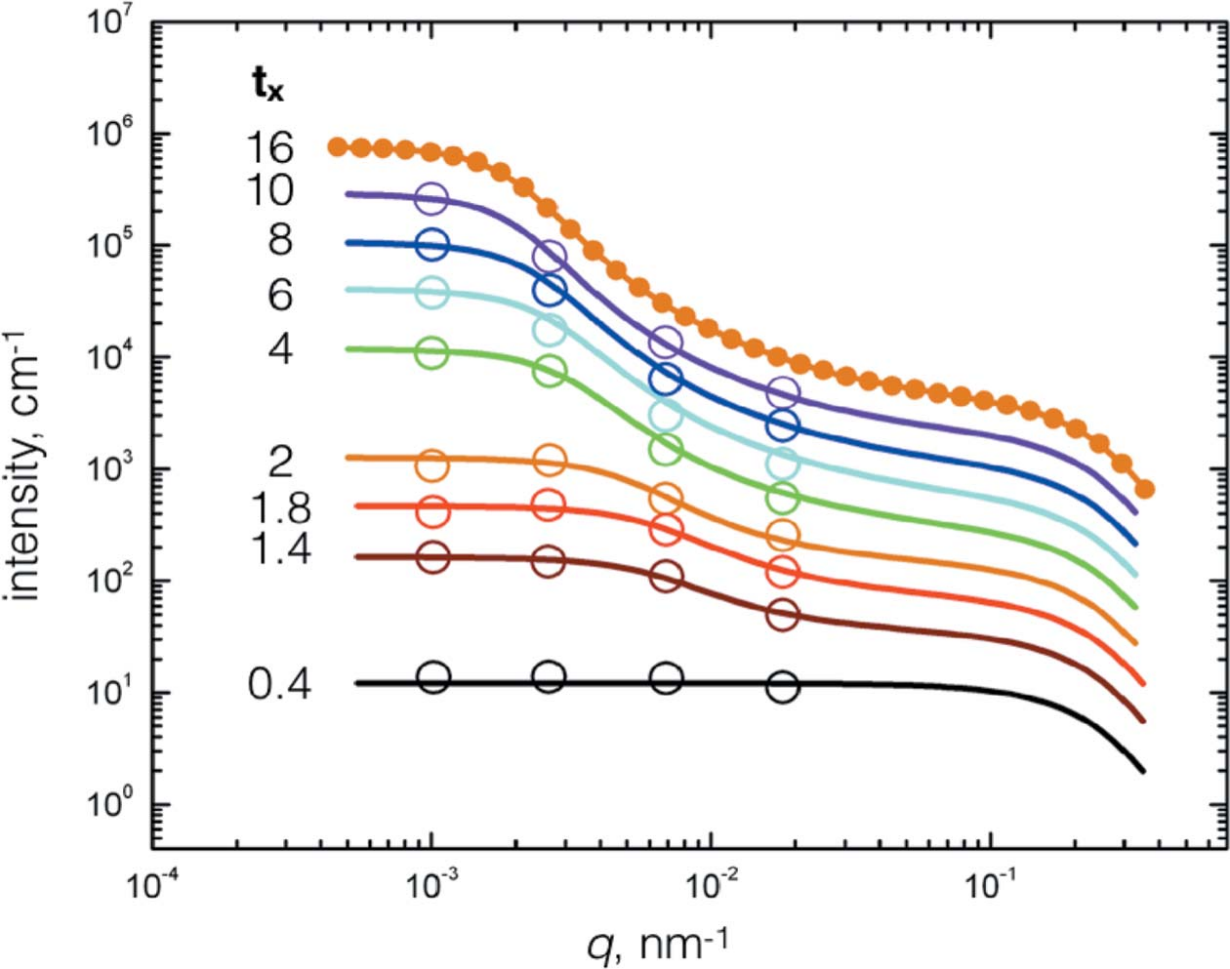
The upper orange curve plot compares the calculated *t_x_* = 16 *I*(*q*) results (line) with the slit-smeared experimental data (circles). Also shown are the USANS *I*(*q*, *t*) calculated intensity curves at reduced times compared with the experimental intensity points at the selected wavevectors listed in Table 1 (open circles). The plots are successively multiplied by a factor of two for clarity. An estimate of the intensity behaviour at short times, the *t_x_* = 0.4 curve, was calculated by simplifying equation (2) to 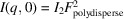 .

The fitting procedure used the Python scripts available within *SASView* (Doucet *et al.*, 2017 ▸) together with resolution functions of slit-smeared USANS to smear the model. For Kookaburra a vertical smearing of 0.0586 Å^−1^ was used to smear the calculated model scattering in *SASView*. *SASView*’s internal version of 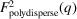 was taken to be a log-normal distribution with a 12% width, and *S*_1_(*q*) was programmed as a C++ *SASView* plugin model with all parameters adjustable. Table 3 ▸ lists the parameter values and the upper orange curve in Fig. 5 ▸ shows the modelled result. The uncertainties listed in Table 3 ▸ are taken from the output of *SASView*’s Levenberg–Marquardt nonlinear fitting algorithm (Levenberg, 1944 ▸; Marquardt, 1963 ▸). These uncertainties estimate local variance in the parameters of this nonlinear fit and do not include propagation of all experimental uncertainties. The experimental uncertainties in the absolute intensity values are a function of *q* but are generally small compared with the uncertainty in the model fitting parameters.

#### 3.6.2. Time-dependent intensity

Time-dependent *I*(*q*, *t*) data were extracted at the *q* values listed in Table 2 ▸ from the SANS and USANS measurements plotted in Fig. 2 ▸ and Figs. 3 ▸(*a*) and 3 ▸(*b*). Because the data overlap for *q* ≳ 5 × 10^−2^ nm^−1^, the *I*(*q*, *t*) data fits were limited to the four USANS wavevectors listed in Table 1 ▸. The run at *t_x_* = 10 was first fitted using the *SASView* approach reported above. The procedure was expedited by seeding with the Table 3 ▸ parameter values of the intensity fit at *t_x_* = 16, but with *I*_2_, α and *r*_sphere_ set as constants (0.05, 0.99538 and 8.31 nm, respectively). Sequential fits for data at earlier times followed the seeding path. Given that the total number of Ludox/SiO_2_ particles is conserved, the increase in low-*q* intensity and the increase in the correlation length are consistent with aggregation particles. This growth in cluster size been discussed in previous work (Muzny *et al.*, 1999 ▸) and the current work represents the quantification of this effect. The resulting time-dependent parameters, which are consistent with the system’s cluster growth and densification, are listed in Table 4 ▸ and the *I*(*q*, *t*) versus *q* plots are displayed in Fig. 5 ▸. To give an estimate of the intensity behaviour at short times, the *t_x_* = 0.4 curve was calculated by simplifying equation (2) to 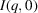 = 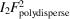 . The calculated *I*(*q*) intensities at *t_x_* = 16 (the upper curve of Fig. 5 ▸) are within ±6% of the experimental values on an absolute scale based on a plot of the residuals. The *I*(*q*, *t*) intensity comparisons are wider because the data are restricted to the intensities at the four selected wavevectors (Table 1 ▸): the percentage ±8% is considered a realistic estimate.

#### 3.6.3. Viscosity

The key parameter ξ from the fit of the SANS and USANS intensities (Table 4 ▸) was used to calculate directly the gelling silica time-dependent viscosities. The kinetic theory viscosity equation for gases is expressed as η ≃ 1/σ^2^, where σ is a characteristic intermolecular distance (McQuarrie, 2000 ▸). By substituting the size ξ for σ we have the general expression

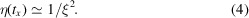

**Figure 6:**
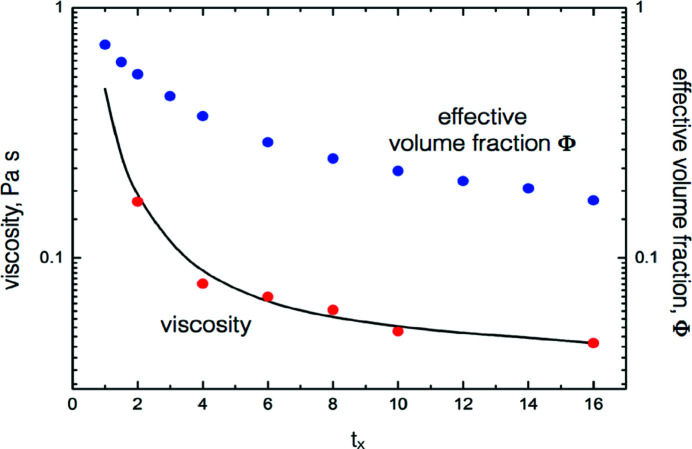
A comparison of the measured viscosities (black line) with the viscosities calculated from equation (4) (red points). The blue points show the effective volume fraction calculated from equation (5).

If the calculated viscosity is scaled by an experimental value at a given time, for example setting η(*t_x_*) to 0.046 Pa s at *t_x_* = 16, the comparative results are as shown in Fig. 6 ▸. [Comparisons for *t_x_* < 2 are excluded because the fitting equations are un­satisfactory, as discussed below, and, in this context, dominated by the form factor at the shorter times.] In short, the viscosity and, as discussed below, the volume fraction and packing density of the shear-influenced silica gel can be calculated directly from a one-parameter fit of the corresponding intensity/wavevector data.

#### 3.6.4. Effective volume fraction: viscosity

The approach here was to estimate the effective volume fraction by coupling with the viscosity. Barnes *et al.* (1989 ▸) defined the effective volume fraction of a material suspended in a liquid as the fraction of the total suspension space occupied by the mater­ial. One can also define a cluster as any entity that is larger than the diameter of the primary object. These definitions generally imply that the material is a solid-like particle. Gillespie (1983 ▸) and other authors used the term ‘effective solids volume fraction’, Φ. Of the many relations proposed to link viscosity with volume fraction, of which de Kruif *et al.* (1985 ▸) and Deepak Selvakumar & Dhinakaran (2017 ▸) are examples, that of Gillespie (1983 ▸) shown in equation (5) has the merit of simplicity:

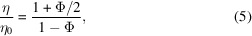

where η_0_ is a normalizing viscosity. The viscosities could be those calculated from equation (4), but experimental values were selected in order to cover a wider range. We set the gelation stable-state viscosity at the reduced time of *t_x_* = 16 (η = 0.0457 Pa s) to the left-hand side of equation (5) and assigned the particle volume fraction ϕ = 0.17 to the right-hand side (Φ); hence η_0_ = 0.0290 Pa s. Overall, therefore, the experimental viscosity and calculated volume fraction pairs follow from equation (5). Table 5 ▸ lists the results displayed in Fig. 6 ▸.

#### 3.6.5. Effective volume fraction: packing density

An alternative and independent calculation of the volume fractions follows by considering the time-dependent cluster change in terms of the gelling silica packing density PD. We surmise that the gel structure develops from that of a very unstructured system to, in principle, that of a solid. One can argue that this growth is reflected in a corresponding increase in the silica packing densities. Dullien (2012 ▸) lists typical descriptions and approximate values for PD: very loose (∼0.5), loose (∼0.6), random close-packed (∼0.64) and dense face-centred cubic (∼0.74), where

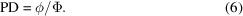




The packing density/Φ relation can be calculated from equation (6) given the known volume fraction of the silica particles (ϕ = 0.17). Shown in Table 5 ▸ are the densities below the maximum packing density (Saha & Bhattacharya, 2010 ▸; Barnes *et al.*, 1989 ▸; de Kruif *et al.*, 1985 ▸) incorporating the volume fractions calculated from the viscosity. As a comparison, values from Dullien (2012 ▸) are also listed with PD = 0.74 assigned to *t_x_* = 10 (the choice was logical because the system is close to its final state at this time). The sets agree well. For the record, shown in Table 5 ▸ are the equivalent effective volume fractions calculated by reversing equation (6) but using the PD of Dullien (2012 ▸).

Table 4 ▸ indicates that the clusters are further apart (ξ increases with time) but scatter more strongly (*I*_1_). Table 5 ▸ also indicates a densification of clusters. Growing silica clusters entrap water from the background solution that is progressively eliminated as the solid coalesces to the final stable state. The overall effective volume fraction can, therefore, be defined (Lewis & Nielsen, 1968 ▸) as

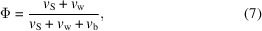

where *v*_S_ is the volume of the solid, *v*_w_ the volume of the trapped water and *v*_b_ the volume of the free solution. Since (*v*_w_ + *v*_b_) is conserved, Φ will decline as *v*_w_ drops. Changes in the relative silica and water contents can be estimated. As an example, a *t*_*x*_ = 6 volume fraction Φ = 0.28 corresponds to a silica volume percentage of 61% with 39% of water, whereas a volume fraction of Φ = 0.18 at *t*_*x*_ ∼ 16 corresponds to 94% silica and 6% water.

## 4. Discussion

### 4.1. Overview

This work has focused on investigating the time-dependent scattering behaviour over a wide wavevector range of a gel-initiated colloidal SiO_2_ system subjected to a steady applied shear rate (250 s^−1^). Fig. 6 ▸ displays the scaled (*t_x_*) scattered intensity/wavevector data compared with the calculations of equations (1) to (3 [Disp-formula fd3]), Section 3.6. The comparison is considered satisfactory for scaled times *t_x_* > 2 and gives an insight into the macroscopic structure of the cluster, for example cluster size, cluster water content, and the variation in the characteristic length ξ and the power law exponent *d*_f_ (Table 4 ▸).

The structure of a gel-initiated silica system is controlled by the growth of clusters of the silica solution particles competing with the formation of a network. In the absence of an applied shear, the network dominates and the effective volume fraction increases to unity at the final gel state (Shih *et al.*, 1990 ▸; Lewis & Nielsen, 1968 ▸). We argue, however, that an applied steady shear breaks the network formation at the time designated as *t*_s_ = 1.0, thus allowing the clusters to densify and grow (Hanley *et al.*, 1999 ▸; Drabarek *et al.*, 2000 ▸; Mohraz & Solomon, 2005 ▸). It was, however, noted that the derivation of the structure factor [equation (3)] accepts the condition that cluster growth is self-similar (Furukawa, 1984 ▸). While the self-similar condition may be satisfied for *t_x_* ≥ 2, the shear-influenced USANS data indicate that it is improbable that the clusters develop a uniform size and/or a uniform structure pattern. It is interesting to recall that a cluster discrepancy has been recognized for many years, for example by Teixeira (1986 ▸), who remarked ‘the ideal scattering law of the fractal object will no longer be obeyed and special treatment of the system cannot be avoided.’ In particular, the issue was addressed by Haw *et al.* (1998 ▸) who gave a definitive summary of the problem. In the work reported here, the plots in Figs. 3 ▸(*a*) and 3 ▸(*b*) show that the scattered micrometre-scale intensity falls at *t_x_* = 1 before sharply rising at *t_x_* ≃ 1.5 and then follows a pattern similar to the nano-scale behaviour at lower wavevectors [Fig. 2 ▸(*c*)]. In fact, the short-time macroscopic behaviour displayed in Fig. 3 ▸(*a*) is characteristic of a floc (Van Dyk & Nakatani, 2013 ▸) not a colloidal cluster.

This apparent contradictory behaviour was considered an opportunity to explore the structure development at early times. The point of departure was to recognize that equation (3) is a phenomenological form representing scattering from a system with fractal-like scatter over a fixed length-scale range and includes correlated scattering at a given size ξ. Correlated scattering at a given length scale is typical for decomposition processes, such as spinodal decomposition, whereas fractal-like scattering is associated with specific growth (aggregation) mechanisms. As mentioned, however, considerable structural development takes place in the range 0.4 < *t_x_* < 1.4.

In order to cover this structural evolution under shear over the full time range of interest, a third term *S*_A_(*q*, *t*), as yet undefined, is introduced into equation (2),

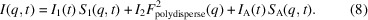

where, at this stage, *I*_1_(*t*), *I*_2_ and *I*_A_(*t*) are fitting parameters. The form factor term 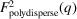 is unaltered and the *S*_1_(*q*, *t*) structure factor has the same form as equation (3) but with the significant difference that the variables have a continuous time dependence,

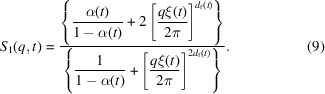




Equation (8) can describe the scattering at all the continuous times of a given run and can therefore fit all the collected data simultaneously, including estimates for consistent values of α(*t*), ξ(*t*), *d*_f_(*t*), *I*_1_(*t*) and *I*_A_(*t*) related to a sample model of *S*_A_(*q*, *t*). To do this, however, the intensity data, measured at well defined wavevectors but at continuous and arbitrary times, has to be slit-smeared accurately. *SASView* (Doucet *et al.*, 2017 ▸) will be used both to accomplish the slit-smearing and to explore possible models for *S*_A_(*q*, *t*).

How best to model *S*_A_(*q*, *t*), and the theoretical underpinning in general, will be first evaluated from non-equilibrium molecular dynamics (NEMD) simulations (Evans & Morris, 1990 ▸) of the gelation process. A precursor has been reported (Butler *et al.*, 1996*a* ▸): the spinodal decomposition simulation of a two-dimensional Lennard–Jones system (Koch *et al.*, 1983 ▸; Barker *et al.*, 1981 ▸) subjected to a liquid quench. It is relevant because of the similarity between the particle aggregation developing after the quench and gel formation.

### 4.2. Length scales

The large neutron beam sizes used here (∼1 cm^2^) can provide a convenient statistical/bulk structural perspective up to 10 µm in size (Sokolova *et al.*, 2019 ▸; Rehm *et al.*, 2018 ▸), but to coordinate a sample’s internal structure over length scales from the nanoscale to the macroscopic is not an easy task. The problem is exacerbated for a system under shear with significant structural changes on the order of minutes. Yet both traditional and recent procedures are limited in their ability to address these problems. For example, X-ray and neutron-based tomography (Zambrano *et al.*, 2019 ▸) are able to provide a three-dimensional perspective on structure, but the measurements for the tomographic reconstruction preclude the required time resolution for this study. Microscopy, including electron microscopy, is a two-dimensional representation but requires careful preparation and sectioning to access a statistically significant reconstruction of three-dimensional structure (Crowther *et al.*, 1970 ▸). Indirect methods (Rueb & Zukoski, 1997 ▸; Shih *et al.*, 1990 ▸; Conrad *et al.*, 2010 ▸; Fluerasu *et al.*, 2007 ▸) where structure is inferred from dynamic measurements, while useful, do not provide the access to direct structural and statistical information inherent in USANS/SANS experiments. There have, however, been relatively recent developments investigating three-dimensional structure over the length scales bridging USANS to the macroscopic scale. We refer to the technique of neutron dark-field imaging, of which a detailed description is given by Harti *et al.* (2017 ▸) and the references therein. The X-ray variant is discussed by Felsner *et al.* (2019 ▸). Experiments using X-ray techniques to investigate the time-dependent behaviour of a system subjected to a shear, or any external force, are anticipated.

## 5. Conclusions

As noted above, while a time development of sheared silica cluster growth can be addressed if *t_x_* > 2, an abrupt change in cluster shape or size, corresponding to a change in viscosity at the un-sheared silica gelation point, is difficult to model. In the work presented here we have initiated a path to lead to a better theoretical insight of the dynamic behaviour of a sheared system out of equilibrium.

On the experimental side, the time-influenced data reported here provide a background for experiments on more complex gels. It has been remarked that dynamic intensity/wavevector measurements are surprisingly uncommon, although there is a solid body of literature on how a gel, or an aggregating system, responds to different applied shear rates.

## Figures and Tables

**Table 1:** USANS points (*q* values) selected to monitor the micrometre-scale time-dependent scattered intensity of the gelling silica system sheared at 250 s^−1^ Points were placed equidistantly on a logarithmic scale to cover a wide *q* range with significant intensity.

Point	*q* value (nm^−1^)
1	1.00 × 10^−3^
2	2.62 × 10^−3^
3	6.87 × 10^−3^
4	1.80 × 10^−2^

**Table 2:** Selected *q* (SANS) for comparison with USANS data

Wavevector	*q* value (nm^−1^)
*q*_5_	4.6 × 10^−2^
*q*_6_	8.7 × 10^−2^
*q*_7_	1.6 × 10^−1^
*q*_8_	3.1 × 10^−1^

**Table:** 

*I*_1_ *S*_1_(*q*)	Fitted parameter	Uncertainty
*I*_1_	6000 × 10^3^ cm^−1^	±10 × 10^3^ cm^−1^
α	0.99538	±0.001
ξ	10.5 µm	±0.7
*d*_f_	2.3643	±0.04

**Table:** 

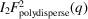	Fitted parameter	Uncertainty
*I*_2_	0.05 cm^−1^	±0.001 cm^−1^
*r*_sphere_	8.31 nm	±0.2 nm
Polydispersity ratio	0.12	±0.01

**Table 4:** Parameters ξ, *d*_f_ and *I*_1_ from the time-dependent *I*(*q*, *t*) data fits for the gelling sample subjected to a constant applied shear rate of 250 s^−1^ The calculation used equations (2) and (3) but with *I*_2_, α and *r*_sphere_ set as constants. The fitted steady-state *I*(*q*) curve (Fig. 4 ▸) is included.

Scaled time *t*_*x*_	Length ξ (µm)	Dimension *d*_f_	Intensity *I*_1_ (×10^−3^ cm^−1^)
16	10.5 ± 0.7	2.4 ± 0.1	6000 ± 10
10	10.0	2.3	4400
8	9.0	2.0	2700
6	8.5	2.1	1800
4	8.0	2.0	900
2	5.5	1.8	100
1.8	4.7	1.7	54
1.4	4.7	1.7	35

**Table 5:** The scaled time path of the experimental viscosity and the calculated effective volume fraction Φ using equation (5) The packing density PD was calculated from equation (6) and compared with the packing densities from Dullien (2012 ▸). Also shown are the effective volume fractions given the PD from Dullien (2012 ▸).

Scaled time *t_x_*	Viscosity *H* (Pa s)	Effective volume fraction Φ using equation (5)	Packing density using equation (6)	Packing density using Dullien (2012 ▸)	Effective volume fraction (PD) Φ using Dullien (2012 ▸)
12	0.0501	0.203			
10	0.0532	0.223	0.76	0.74	0.23
8	0.0578	0.25	0.68	0.64	0.26
6	0.0656	0.29	0.59	0.6	0.28
4	0.0862	0.369	0.46	0.5	0.34
3	0.114	0.443	0.38		
2	0.175	0.542	0.31		
1.5	0.243	0.606	0.28		
1	0.474	0.712	0.24		

## Footnotes

1 Trade names are provided only to specify procedures adequately and do not imply endorsement by the National Institute of Standards and Technology. Similar products by other manufacturers may be found to work as well or better.
